# Fate of *Cryptosporidium* and *Giardia* through conventional and compact drinking water treatment plants

**DOI:** 10.1007/s00436-023-07947-8

**Published:** 2023-08-26

**Authors:** Ahmed S. Moussa, Ameen A. Ashour, Mohammad I. Soliman, Hoda A. Taha, Ahmad Z. Al-Herrawy, Mahmoud Gad

**Affiliations:** 1 Reference Laboratory, Drinking Water and Wastewater Holding Company, Cairo, Egypt; 2 Zoology Department, Faculty of Science, Ain Shams University, Cairo, Egypt; 3 Environmental Parasitology Laboratory, Water Pollution Research Department, National Research Centre, 12622, Dokki, Giza, Egypt

**Keywords:** Enteric protozoa parasites, Drinking water treatment plants, Modern techniques

## Abstract

Over the past three decades, a notable rise in the occurrence of enteric protozoan pathogens, especially *Giardia* and *Cryptosporidium* spp., in drinking water sources has been observed. This rise could be attributed not only to an actual increase in water contamination but also to improvements in detection methods. These waterborne pathogens have played a pivotal role in disease outbreaks and the overall escalation of disease rates in both developed and developing nations worldwide. Consequently, the control of waterborne diseases has become a vital component of public health policies and a primary objective of drinking water treatment plants (DWTPs). Limited studies applied real-time PCR (qPCR) and/or immunofluorescence assay (IFA) for monitoring *Giardia* and *Cryptosporidium* spp., particularly in developing countries like Egypt. Water samples from two conventional drinking water treatment plants and two compact units (CUs) were analyzed using both IFA and qPCR methods to detect *Giardia* and *Cryptosporidium*. Using qPCR and IFA, the conventional DWTPs showed complete removal of *Giardia* and *Cryptosporidium*, whereas Mansheyat Alqanater and Niklah CUs achieved only partial removal. Specifically, *Cryptosporidium* gene copies removal rates were 33.33% and 60% for Mansheyat Alqanater and Niklah CUs, respectively. Niklah CU also removed 50% of *Giardia* gene copies, but no *Giardia* gene copies were removed by Mansheyat Alqanater CU. Using IFA, both Mansheyat Alqanater and Niklah CUs showed a similar removal rate of 50% for *Giardia* cysts. Additionally, Niklah CU achieved a 50% removal of *Cryptosporidium* oocysts, whereas Mansheyat Alqanater CU did not show any removal of *Cryptosporidium* oocysts. Conventional DWTPs were more effective than CUs in removing enteric protozoa. The contamination of drinking water by enteric pathogenic protozoa remains a significant issue globally, leading to increased disease rates. Infectious disease surveillance in drinking water is an important epidemiological tool to monitor the health of a population.

**Supplementary Information:**

The online version contains supplementary material available at 10.1007/s00436-023-07947-8.

## Introduction

Water-related diseases are responsible for the highest number of deaths and diseases worldwide, according to the World Health Organization (WHO), with more than 3.4 million fatalities each year. Children account for around 1.4 million of these deaths (Abuseir 2023). In terms of causing fatalities, the lack of clean and safe drinking water along with inadequate sanitation surpasses war, terrorism, and weapons of mass destruction combined (Abuseir 2023). Water-related illnesses, such as gastrointestinal infections, diarrhea, and systemic diseases, lead to an annual economic loss of approximately US$ 12 billion globally (Alhamlan et al. 2015). Protozoan parasites like *Cryptosporidium* spp., and *Giardia* spp., are from the most frequently identified as the cause of diarrheal outbreaks in both developed and developing countries (Karanis et al. 2007; Gad et al. 2019; Al-Rifai et al. 2020). The right to access safe water, which is essential for the survival of living beings, has been acknowledged as a universal human right (Taviani et al. 2022). Consumption or exposure to contaminated water can lead to the transmission of various pathogens, including enteric bacteria, viruses, and parasites, causing severe diseases that pose a significant global public health concern (WHO 2019). So, it is crucial to monitor drinking water for pathogens to protect human and animal health.

Waterborne outbreaks caused by protozoa contamination are a significant concern. *Cryptosporidium* and *Giardia* species are particularly notable as they can survive in aquatic environments despite the use of chlorine disinfectants (Elmehy et al*.* 2021). Regrettably, many people worldwide do not have access to safe water, which is free from harmful pathogens and contaminants. This issue is a significant public health concern, even for developed countries (McKee and Cruz 2021). Protozoan parasitic diseases transmitted through contaminated water are found globally and have caused both epidemic and endemic infections in developing countries (Cotruvo et al. 2004; Baldursson and Karanis 2011). For the past four decades, there has been a significant global focus on *Cryptosporidium* and *Giardia* species due to their ability to cause waterborne and foodborne illnesses (Mahmoudi et al. 2017; Rosado-García et al. 2017). These protozoa are excreted through feces and have a remarkable resistance to environmental factors, such as high temperatures, chemical water disinfectants, and dehydration (King and Monis 2007).

In 2012, the prevalence of giardiasis in Europe was 5.4 cases per 100,000 population. As for cryptosporidiosis, the prevalence was 10.5 for females and 13.8 for males (ECDC 2014). Unfortunately, there is a lack of comprehensive data on the prevalence of cryptosporidiosis and giardiasis in Egypt and only a limited number of environmental studies focusing on *Cryptosporidium* and *Giardia* have been identified (El-Kowrany et al. 2016; Gad et al. 2019; Rizk et al. 2019; Hamdy et al. 2019). The main objectives of this study were (1) to assess the efficacy of different drinking water treatment methods in removing *Cryptosporidium* and *Giardia* parasites, which pose risks to human health, (2) to quantify the concentration of these parasites using qPCR and IFA detection methods, both of which were effective in identifying *Cryptosporidium* and *Giardia* in water samples, and (3) to investigate the prevalence of these parasites in both raw and treated water samples.

## Material and methods

### Sampling and DWTPs descriptions

**Fig. 1:**
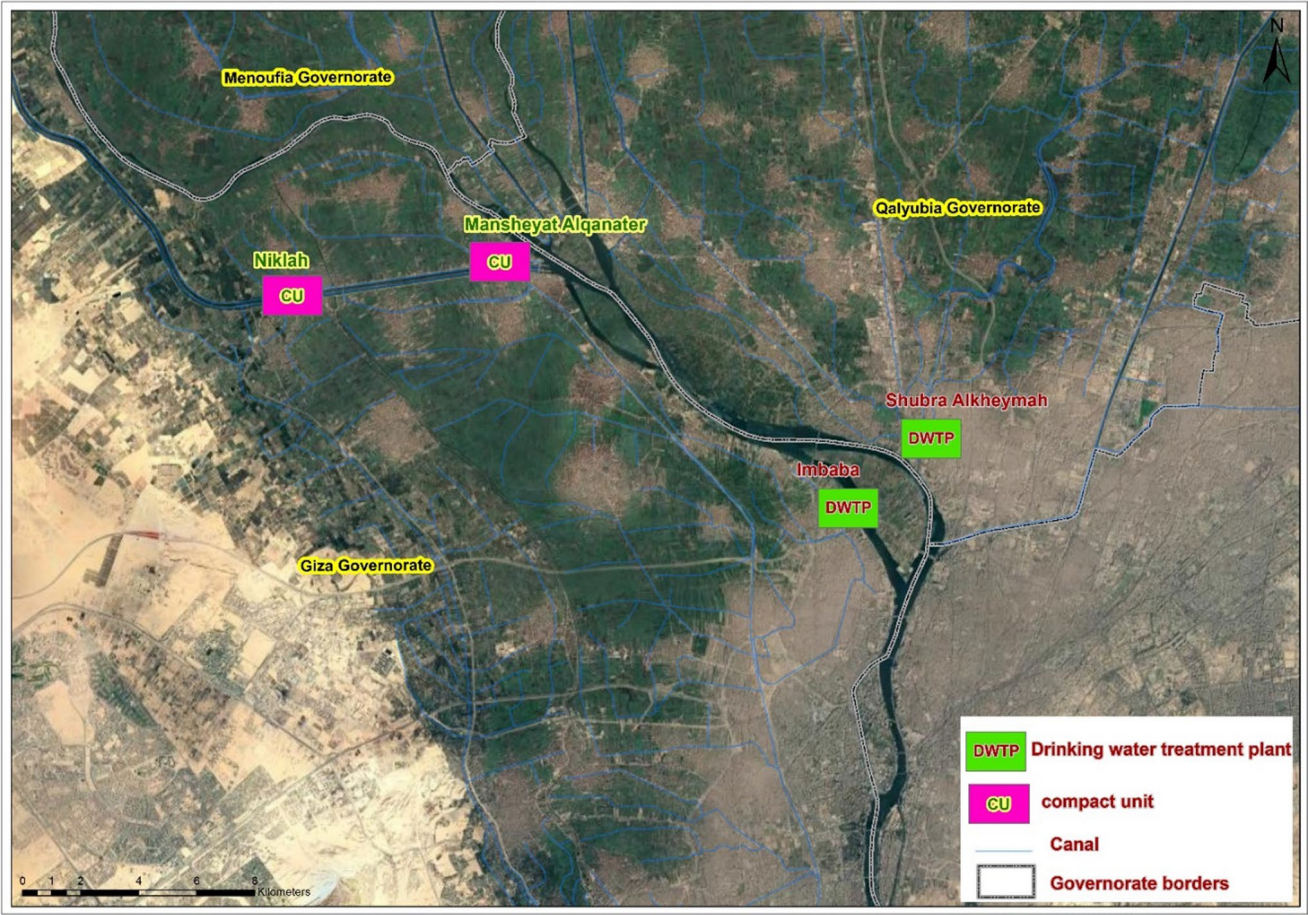
Location of the four examined drinking water treatment plants

For the present study, four DWTPs were selected. Among them, two were conventional DWTPs catering to large city communities, while the other two were small CUs serving comparatively smaller communities (Fig. 1). The two conventional DWTPs are Shubra Alkheymah, which produces 46300 m^3^ of drinking water per day and serves 2.5 million persons, and is located in the Shubra Alkheymah district. Shubra Alkheymah DWTP is composed of an intake system that is supplied with raw surface water from the mainstream of the Nile River, a distribution well, 12 clarifiers (working with a pulsator), 34 rapid sand filters, and a drinking water storage tank. The second conventional plant is Imbaba DWTP, which produces 1.43 million m^3^ of drinking water per day and serves people in Al-Remayah, Al-Baragil, Al-Khalayfa, Nahia, Ezbet Al-Eseely, Imbaba, Al-Waraq, and Al-Kitkat districts. The intake of Imbaba DWTP is supplied with raw surface water from the mainstream of the Nile River. Imbaba DWTP is composed of an intake system, distribution well, 20 clarifiers (working with a pulsator), 80 rapid sand filters, and a drinking water storage tank (Fig.S1).

The study also included two drinking water treatment compact units (CUs): Niklah CU and Mansheyat Alqanater CU. Niklah CU has a daily drinking water production capacity of 2040 m^3^, and its freshwater supply is sourced from El-Nasery canal, which branches off from the Nile River. Mansheyat Alqanater CU, on the other hand, produces 2000 m^3^ of drinking water per day and is continuously supplied with fresh water from El-Behery canal, which also branches off from the Nile River. Both Niklah and Mansheyat Alqanater CUs share a similar design, consisting of an intake system and a compact unit where clarification and sand filtration processes take place. The treatment process concludes with the final product being stored in a drinking water tank (Fig. S2).

A total of 96 samples were collected from the inlet (raw freshwater) and outlet (final treated drinking water) of two conventional DWTP (i.e., Shubra Alkheymah and Imbaba) and two CUs (i.e., Niklah and Mansheyat Alqanater) within Greater Cairo, Egypt (Fig. 1). Each sample was collected in duplicate, one (10 L volume) of them was used for IFA technique and the other one (10 L volume) for qPCR technique. The samples were collected monthly at the same time from each sampling site for 1 year from February 2019 to January 2020.

### Immunofluorescence assay

Each sample was filtered through sterile nitrocellulose membranes (142 mm diameter and 0.8μm pore size) using a sterilized stainless steel pressure filtration system (Millipore). The nitrocellulose membrane filter was removed from the filter housing and transferred into a suitable clean glass Petri dish (142mm diameter). About 25mL of eluent (0.1% Tween 80) was gently poured on the surface of the membrane filter to facilitate the detachment of particulate material from the membrane (ISO/FDIS 15553:^2006^). The last step was repeated and the obtained washing solution was subjected to centrifugation at 1500 ×g for 15min. The supernatant was aspirated and the obtained pellet was resuspended in 10 mL phosphate buffer saline (pH = 7.4), vortexed from 10 to 15 s, and transferred into a Leighton tube. One milliliter of the 10X SL-buffer-A and 1 mL of the 10X SL-buffer-B were added to a Leighton tube. Then, 100 μL of the resuspended Dynabeads *Cryptosporidium* and 100 μL of the resuspended Dynabeads *Giardia* (Dynabeads™ GC-Combo, ThermoFisher Scientific, USA) were added to the solution in Leighton tube. The immunomagnetic separation and immunostaining (DAPI and FITC) steps were conducted according to EPA Method 1623 (2005) and ISO/FDIS 15553:2006. The positive controls for both parasites (AccuSpike™-IR, Waterborne™, Inc., USA) were used along with each batch of samples for confirming the productivity of the method.

### Quantitative real-time PCR assay

Similar concentration steps to IFA until obtaining the solution from the dissociation of the Dynabeads-cysts/oocysts complex were applied for qPCR. The immunomagnetic purified samples were subjected to extraction of environmental DNA using the DNeasy PowerLyzer PowerSoil Kit (QIAGEN, USA) according to the manufacturer’s instructions. The uniplex qPCR assay was performed to quantify the target protozoa in the samples; a qPCR reaction was performed in a 20-μL reaction volume using a QuantiNova syber green qPCR kit (Qiagen, Germany). The reaction mixture was composed of 5 μL of the DNA template, 10 μL of the master mix, 0.5 μL from each primer (forward and reverse) for *Cryptosporidium* (Haque et al. 2007) and *Giardia intestinalis* (Guy et al. 2003), and 4 μL of Nuclease-free water. The PCR temperature conditions were 95°C for 10 min and 45 cycles of 15 s at 95°C and 1 min at 60°C. Nuclease-free water was also included in each run as a negative control. Absolute quantification of gene copy (GC) was performed by comparing cycle threshold (Ct) values to the DNA standard, which was included in every qPCR run (BIO-RAD, CFX96 Rial-Time System, USA). The DNA standards for *Giardia* and *Cryptosporidium* were prepared from AccuSpike-IR (Waterborne™, Inc., USA). The DNAs were extracted using the DNeasy PowerLyzer PowerSoil Kit (QIAGEN, USA). The PCR reactions were performed separately for both *Giardia* and *Cryptosporidium*. The obtained PCR products were purified using GeneJET PCR Purification Kit (Thermo Scientific, Lithuania). Nucleic acid concentrations of the purified PCR products were determined by NanoDrop Fluorospectrometer (Thermo-Scientific, USA). The number of DNA copies was determined by multiplying the DNA concentration by Avogadro’s constant and dividing by the product size and average weight of a base pair as in the following website https://cels.uri.edu/gsc/cndna.html (Rizk and Hamza (2021). The standard curves of each examined organism were separately prepared by tenfold serial dilution of the nucleic acid standard ranging from 5×10^1^ to 5×10^6^ copies/reaction. The limits of detection for the assay were determined as more than or equal to 10 GC/reaction.

### 18S rRNA high-throughput amplicon sequencing analysis

The DNAs of eight samples were selected randomly to cover the inlet and outlet of different DWTPs and CUs during the course of the study. The hypervariable V4 region of eukaryotic 18S rRNA genes was amplified by using A-528F (5′- GCGGTAATTCCAGCTCCAA-3′) and B-706R (5′- AATCCRAGAATTTCACCTCT-3′) primer pair (Cheung et al. 2010). The PCR amplification cycles consisted of initial denaturation at 95°C for 5 min, followed by 25 cycles of 95°C for 30 s, 50°C for 30 s, and 72°C for 60 s, and a final extension at 72°C for 10 min. The purified PCR products were sequenced using an Illumina platform (Illumina Inc., San Diego, CA, USA).

### Sequence analysis

The raw paired-end reads underwent denoising and assembly using DADA2 v1.16.0 (https://benjjneb.github.io/dada2/tutorial.html) (Callahan et al. 2016). Amplicon sequence variants (ASVs) were generated at 100% sequence identity from the high-quality reads. The taxonomic classification of 18S rDNA reads was conducted using RDP classifier and PR^2^ database (Guillou et al. 2013).

### Statistical analysis

The Kruskal-Wallis test was employed to examine the seasonal variation of *Giardia* and *Cryptosporidium* in the surface water (intakes of the DWTPs and CUs). Significance was attributed to *P* values below 0.05. Statistical analyses and visualization were performed using Origin (Pro) 2021, PRIMER v.7.0.21, and R v4.2.2 (https://www.r-project.org/).

## Results

### Morphological characteristics of Giardia cysts and Cryptosporidium oocysts using IFA

**Fig. 2:**
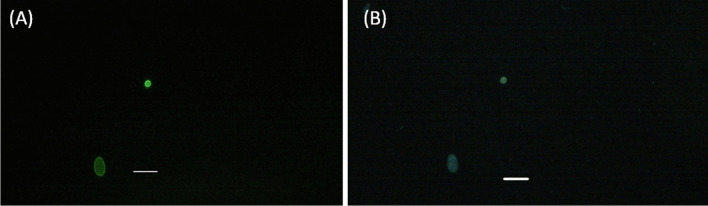
Photomicrograph of **A** *Giardia* cyst and *Cryptosporidium* oocyst stained with FITC stain. **B** *Giardia* cyst and *Cryptosporidium* oocyst stained with DAPI stain under a fluorescent microscope. Bar = 20μm

The positive samples containing *Giardia* cysts and *Cryptosporidium* oocysts were easily distinguishable through their apple-green color when stained with an immune-fluorescent stain (FITC). The oval shape cyst wall of the *Giardia* cysts made them easily recognizable, and each cyst contained 2–4 nuclei that were identifiable by DAPI stain. The size of the detected *Giardia* cysts ranged from 8–18 × 5–15μm (as shown in Fig. 2). Similarly, the rounded shape oocyst wall of *Cryptosporidium* oocysts made them easily detectable, and each oocyst contained 4 sporozoites that were visible with DAPI stain. The detected oocysts had a diameter of 4–6μm (Fig. 2).

### Occurrence and removal of *Giardia* and *Cryptosporidium *in DWTPs and CUs using IFA

The immunofluorescence stain detected *Giardia* cysts in 11.5% of all collected water samples (both raw and treated), while *Cryptosporidium* cysts were detected in 18.8% of the samples (Table S1). In the inlets of conventional DWTPs (Imbaba and Shubra Alkheymah), the prevalence of *Giardia* cysts ranged from 8.3 to 33.3%, while it was 16.7% in the inlets of CUs (Mansheyat Alqanater and Niklah). No *Giardia* cysts were detected in the final treated drinking water of Imbaba and Shubra Alkheymah DWTPs. However, the final treated drinking water of Mansheyat Alqanater and Niklah CUs was contaminated by 8.3% of *Giardia* cysts (Table 1). Furthermore, higher prevalence rates of *Cryptosporidium* oocysts were observed in the inlets of both conventional DWTPs (range: 25–41.7%) and CUs (16.67–33.3%). *Cryptosporidium* cysts were detected in 16.7% of CUs outlet water samples, while none was found in conventional DWTPs outlet samples (Table 1).

**Table 1:** Occurrence of *Giardia* cysts and *Cryptosporidium* oocysts in the examined DWTPs and Compact units using IFA and qPCR

Station	*Giardia* cysts				*Cryptosporidium* oocysts			
	Raw water samples		Treated water samples		Raw water samples		Treated water samples	
	IFA (%)	qPCR (%)	IFA (%)	qPCR (%)	IFA (%)	qPCR (%)	IFA (%)	qPCR (%)
Imbaba DWTP	33.3	33.3	0	0	25	25	0	0
Shubra DWTP	8.3	8.3	0	0	41.7	41.7	0	0
Mansheyt Alqanater CU	16.7	16.7	8.3	16.7	16.7	25	16.7	16.7
Niklah CU	16.7	16.7	8.3	8.3	33.3	41.7	16.7	16.7

**Fig. 3:**
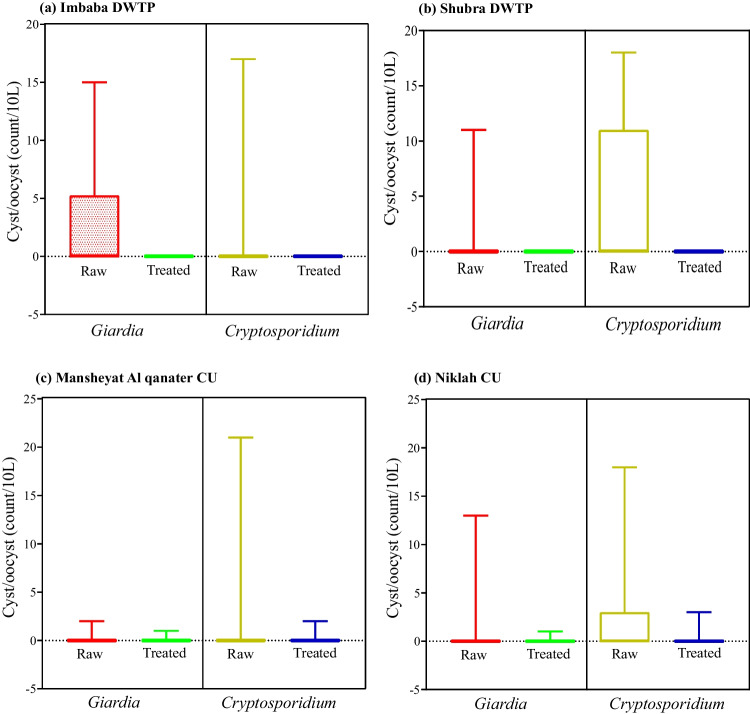
Box plot shows the minimum and maximum concentrations (cysts/oocysts) of *Giardia* and *Cryptosporidium* by IFA in the examined DWTPs (Shubra Alkheymah and Imbaba) and CUs (Mansheyat Alqanater and Niklah)

The maximum number of *Giardia* cysts recorded in inlets of Imbaba DWTP was 15 cysts/10L, followed by 13 cysts/10L, 11 cysts/10L, and 2 cysts/10L in Niklah CU, Shubra Alkheymah DWTP, and Mansheyat Alqanater CU, respectively. However, the maximum number of *Cryptosporidium* oocysts was recorded in inlets of Mansheyat Alqanater CU (21 oocysts/10L), Niklah CU (18 oocysts/10L), Shubra Alkheymah DWTP (18 oocysts/10L), and Imbaba DWTP (17 oocysts/10L). Few oocysts/cysts (≤ 3) were detected in the outlets of the CUs and no oocysts/cysts were found in the outlets of the conventional DWTPs (Fig. 3). Using IFA, both Mansheyat Alqanater and Niklah CUs showed a similar removal rate of 50% for *Giardia* cysts. Additionally, Niklah CU achieved a 50% removal of *Cryptosporidium* oocysts, whereas Mansheyat Alqanater CU did not show any removal of *Cryptosporidium* oocysts (Table 2).

**Table 2:** Removal of *Giardia* and *Cryptosporidium* in DWTPs and CUs by qPCR and IFA

Technique	Station	*Giardia*			*Cryptosporidium*		
		Raw water (+ve)	Treated water (+ve)	Removal (%)	Raw water (+ve)	Treated water (+ve)	Removal (%)
qPCR	Imbaba DWTP	4	0	100	3	0	100
	Shubra Alkheymah DWTP	1	0	100	5	0	100
	Mansheyat Alqanater CU	2	2	0	3	2	33.33
	Niklah CU	2	1	50	5	2	60
IFA	Imbaba DWTP	4	0	100	3	0	100
	Shubra Alkheymah DWTP	1	0	100	5	0	100
	Mansheyat Alqanater CU	2	1	50	2	2	0
	Niklah CU	2	1	50	4	2	50

### Occurrence and removal of *Giardia* and *Cryptosporidium* in DWTPs and CUs using qPCR

The qPCR analysis detected *Giardia* in 12.5% of all collected water samples (both raw and treated). Additionally, the qPCR analysis identified *Cryptosporidium* in 20.83% of all collected water (Table S1). More data have been provided in Table S2. The number of positive samples for *Giardia* was higher in the inlets of Imbaba DWTP (*n* = 4) compared to other drinking water plants (*n* = 1 or 2). However, the inlets of Niklah CU and Shubra Alkheymah DWTP had more positive samples for *Cryptosporidium* (*n* = 5 for each) compared to Mansheyt Alqanater CU and Imbaba DWTP (*n* = 3 for each). Overall, it was observed that conventional DWTPs showed higher efficiency in removing *Giardia* and *Cryptosporidium* compared to CUs. Both *Giardia* and *Cryptosporidium* removal percentages reached 100% in Shubra Alkheymah and Imbaba DWTPs. In contrast, Mansheyat Alqanater and Niklah CUs achieved *Cryptosporidium* removal percentages of 33.3% and 60%, respectively (Table 2).

In the inlets of conventional DWTPs (Imbaba and Shubra Alkheymah), the prevalence of *Giardia* genes ranged from 8.3 to 33.3%, while it was 16.7% in the inlets of CUs (Mansheyat Alqanater and Niklah). No *Giardia* genes were detected in the final treated drinking water of Imbaba and Shubra Alkheymah DWTPs. However, a similar occurrence percentage (16.7%) of *Giardia* genes was found in the final treated drinking water of Mansheyat Alqanater and Niklah CUs (Table 1). Furthermore, higher prevalence rates of *Cryptosporidium* genes were observed in the inlets of both conventional DWTPs (range: 25–41.7%) and compact units (25–41.7%). *Cryptosporidium* genes were detected in 16.7% of CUs outlet water samples, while none was found in conventional DWTPs outlet samples (Table 1).

**Fig. 4:**
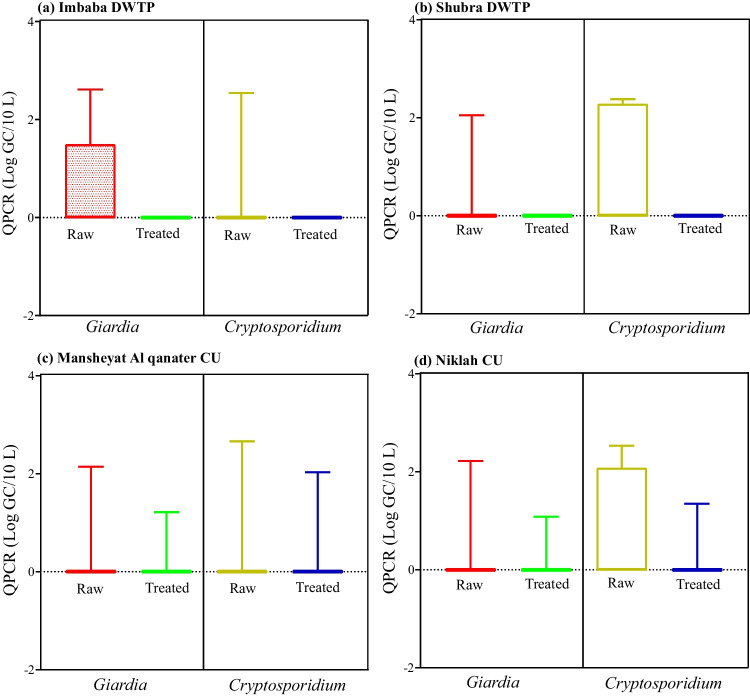
Box plot shows the minimum and maximum concentrations (Log) of *Giardia* and *Cryptosporidium* by qPCR in raw and treated water samples of the examined DWTPs (Shubra Alkheymah and Imbaba) and CUs (Mansheyat Alqanater and Niklah)

The counts of *Giardia* and *Cryptosporidium* genes in the inlet water samples of conventional DWTPs ranged 0–2.61 GC/10L and 0–2.54 GC/10L, respectively. *Giardia* and *Cryptosporidium* were not detected in the outlet water samples of Imbaba and Shubra Alkheymah DWTPs. The maximum concentration of *Giardia* and *Cryptosporidium* was 2.2 GC/10L and 2.6 GC/10L, respectively, in the inlet water samples of the CUs, while the maximum concentration of the same parasites in the outlet water samples of the CUs was 1.17 and 2.0 GC/10L, respectively (Fig. 4). No seasonal variations for *Giardia* and *Cryptosporidium* (Kruskal-Wallis test: *P* > 0.05) in surface water samples were observed.

### 18S rRNA amplicon sequencing

**Fig. 5:**
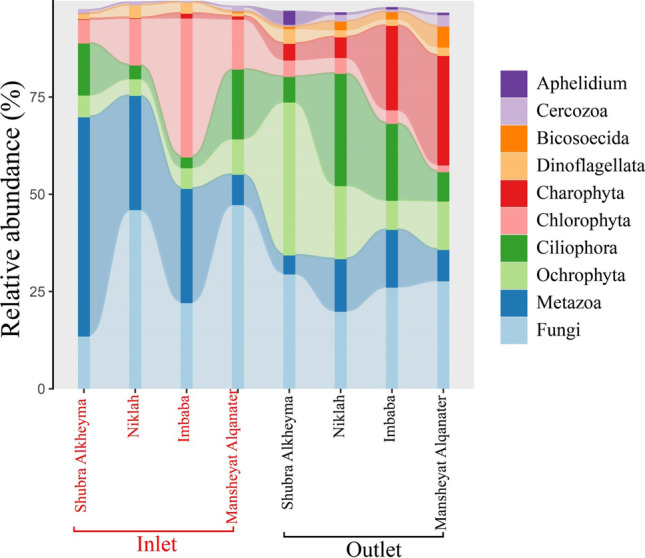
Relative abundance of top 10 microeukaryotic taxa at Rank 3 in the DWTPs and CUs

**Fig. 6:**
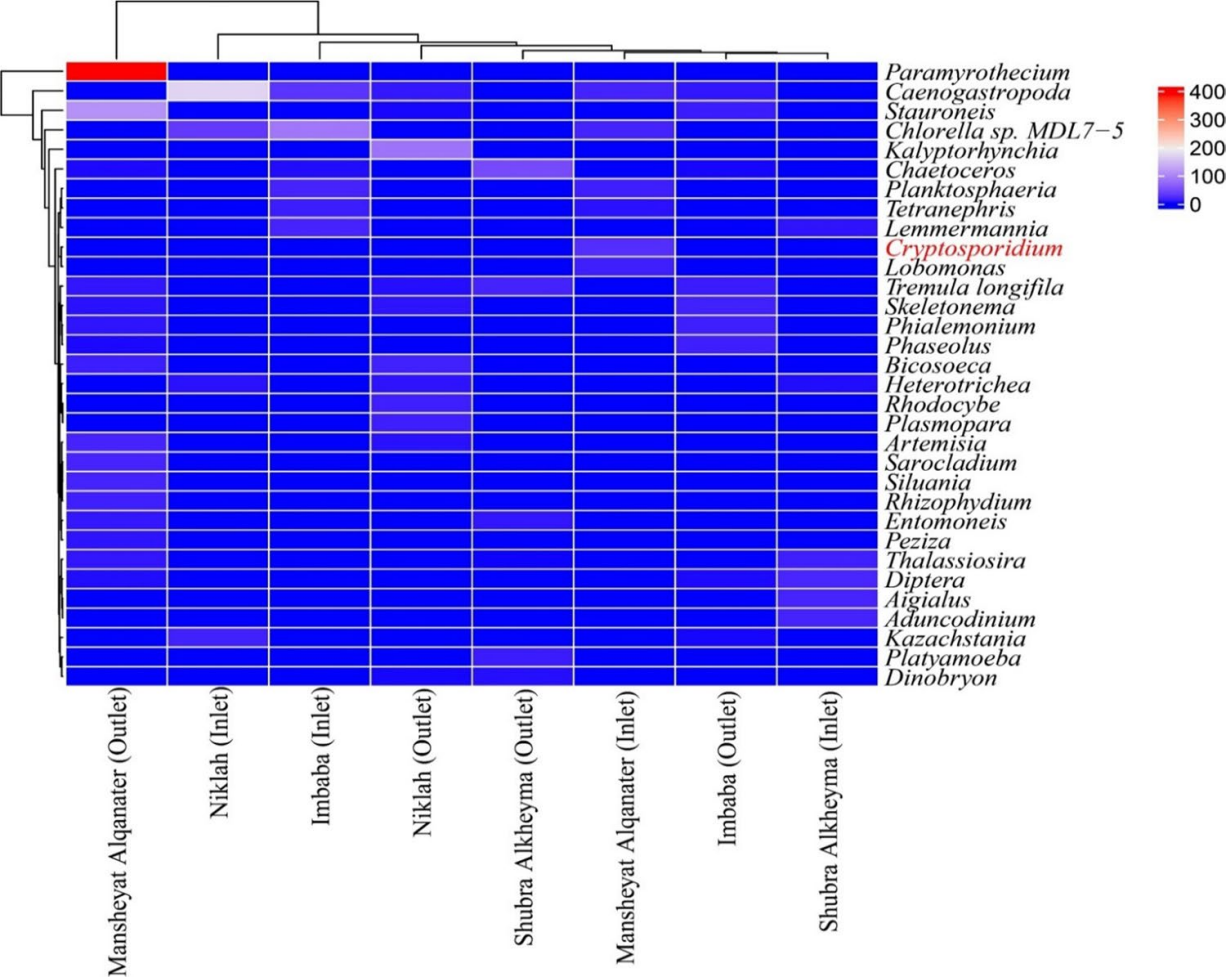
Heatmap illustrating the abundance of select rare microeukaryotic genera in the DWTPs and CUs. Color gradient ranges from blue (low abundance) to red (high abundance). Samples were collected from inlet and outlet of CUs (Niklah and Mansheyat Alqanater) and DWTPs (Imbaba and Shubra Alkheyma)

A total of 3447 microeukaryotic ASVs were detected in inlets and outlets of the DWTPs and CUs using 18S rRNA next-generation sequencing. The barplot showed the relative abundance of the top ten microeukaryotic taxa at Rank 3. Several taxa groups (e.g., Ciliophora, Cercozoa) revealed a significant abundance in the inlets and outlets of the DWTPs and CUs. Fungi and Metazoa were the most abundant taxa groups (≥ 13.6%) and (≥ 4.8%) in different DWTPs and CUs stages, respectively. Lower abundant taxa such as Apicomplexa is not shown in Fig. 5. From the lower abundant taxa, *Cryptosporidium* was detected in the inlet of Mansheyat Alqanater CU (Fig. 6). Several other pathogenic or potentially pathogenic genera (e.g., *Blastocystis* and *Vermamoeba*) were detected using 18S rRNA amplicon sequencing.

## Discussion

DWTPs employ a combination of physical, chemical, and biological processes to remove contaminants from source water. The effectiveness of these processes in eradicating *Cryptosporidium* and *Giardia*, two common waterborne protozoan parasites, relies on several factors, such as the parasite size, treatment method employed, and operational conditions of the plant. Research has shown that *Cryptosporidium* and *Giardia* can be effectively removed through conventional DWTPs that include filtration and disinfection steps. However, the removal efficiency varies depending on the specific treatment process and the operational conditions. These findings align with the results of a previous study conducted in Egypt, where the removal percentages of *Giardia* and *Cryptosporidium* by conventional DWTPs were also reported as 100% (Ali et al. 2004). In Malaysia, the conventional DWTP demonstrated a removal rate of 92.9% for *Giardia* and 100% for *Cryptosporidium* (Richard et al. 2016). Similarly, in Southern Brazil, the DWTP, which encompasses a comprehensive water treatment cycle, achieved a 100% removal rate for both *Giardia* and *Cryptosporidium* (Almeida et al. 2015). In a Spanish investigation, conventional DWTPs achieved a 100% removal rate for both *Cryptosporidium* and *Giardia* (Carmena et al. 2007). On the contrary, a study conducted in China reported a lower removal percentage (66.7%) for *Giardia* through conventional DWTPs, while no removal was observed for *Cryptosporidium* (Kui et al. 2021). Regular monitoring of these rates is crucial to ensure the effectiveness of the treatment processes in eliminating parasites from the water and delivering safe drinking water to consumers.

Less information is available on the removal rates of *Cryptosporidium* and *Giardia* through CUs for drinking water treatment. CUs are typically designed to treat smaller volumes of water and are commonly utilized in remote or rural areas where conventional water treatment methods may not be accessible (Al-Herrawy and Gad 2017; Ali et al. 2004). In Egypt, the researchers found *Cryptosporidium* and *Giardia* in the inlet water samples of the two CUs only (Ali et al. 2004). In the present study, the removal rate of *Giardia* and *Cryptosporidium* in the CUs reached up to 60%. Similarly, a study on Spanish CUs with a comparable structure reported a removal rate of 64.3% for *Giardia*, while *Cryptosporidium* was not effectively removed by the system (Carmena et al. 2007). The removal rates of *Cryptosporidium* and *Giardia* in conventional drinking water treatment plants (DWTPs) can fluctuate depending on several factors, such as changes in the source water quality, variations in the plant operating conditions, and the effectiveness of treatment processes. Compared to conventional DWTPs, some compact units (CUs) may exhibit lower parasite removal rates due to their smaller size or limited treatment capacity.

In the current research, the average prevalence of *Cryptosporidium* and *Giardia* in the Nile River (intake water samples) was 33.3% and 20.8%, respectively. Similar findings were reported for *Cryptosporidium* prevalence in the Nile River, Egypt (33% by direct microscopy) (El-Khayat et al. 2022) and in raw water samples in Iran (30% by IFA) (Mahmoudi et al. 2013). The higher prevalence rate for *Giardia* in raw water samples collected from the Quindío River basin was reported in Colombia (43.6% by PCR) (Pinto-Duarte et al. 2022), for *Cryptosporidium* and *Giardia* in Greece (> 47% by IFA) (Ligda et al. 2020), *Giardia* cysts and *Cryptosporidium* oocysts in Canadian rivers (> 63% by IFA and > 50% by PCR) (Prystajecky et al. 2014). However, a lower prevalence of *Giardia* cysts in Ethiopian rivers (16% by IFA) was recorded (Kifleyohannes and Robertson 2020).

In the present study, the concentration of *Giardia* cysts in raw water ranged from 0 to 15 cysts/10L, while the *Cryptosporidium* concentration in raw water ranged from 0 to 21 oocysts/10L. Other studies have reported different concentration ranges for *Cryptosporidium* and *Giardia* in raw water samples from various regions. For instance, in Greece, the *Cryptosporidium* oocyst concentration ranged from 0 to 0.9 oocysts/10L, and the *Giardia* cyst concentration ranged from 0 to 4.3 cysts/10L (Ligda et al. 2020). In Iran, the *Giardia* cyst concentration ranged from 1 to 1800 cysts/10L (Mahmoudi et al. 2013), and in Taiwan, the *Giardia* cyst and *Cryptosporidium* oocyst concentration in raw water samples ranged from 0.2 to 31.2 cysts/10L and from 0.23 to 80.14 oocysts/10L, respectively (Hsu et al. 1999). In Ethiopia, the *Giardia* cyst and *Cryptosporidium* oocyst concentration in raw water samples ranged from 3 to 22 cysts/10L and from 1 to 3 oocysts/10L, respectively (Kifleyohannes and Robertson 2020). Therefore, it is important to monitor water quality regularly and choose a treatment technology that is appropriate for specific conditions.

In the present study, no significant seasonal variations were observed for *Giardia* and *Cryptosporidium* in the surface water. A previous study conducted in Colombia (Escobar et al. 2022) also reported that seasonality did not have a strong impact on the occurrence of *Cryptosporidium* and *Giardia* in drinking water systems. In contrast, a study conducted in Egypt (Hamdy et al. 2019) found evidence of seasonality in the prevalence of both *Giardia* and *Cryptosporidium* cysts in tap water, with higher levels observed during the summer season. The prevalence and concentration of *Cryptosporidium* and *Giardia* in source waters can also vary widely depending on the location, season, sample volume, and detection methods employed. One potential limitation of this study was the use of membrane filters as the concentration method instead of cartridge filtration, which may have influenced the prevalence and concentration of *Cryptosporidium* and *Giardia* detected in the water samples. In a previous study by Wohlsen et al. (2004), various filtration methods including Pall Life Sciences Envirochek (EC) standard filtration and Envirochek high-volume (EC-HV) membrane filters, the Millipore flatbed membrane filter, the Sartorius flatbed membrane filter (SMF), and the Filta-Max (FM) depth filter were evaluated for recovery of *Cryptosporidium parvum* oocysts and *Giardia lamblia* cysts. The EC-HV membrane filter (EC-HV-R) showed the highest range of recovery rates for both oocysts (36–76%) and cysts (44–72%), followed by the FM depth filter (oocysts: 18–39%; cysts: 30–63%) and SMF (oocysts: 12–19%; cysts: 32–40%) (Wohlsen et al. 2004). However, a contrasting study conducted by Hsu et al. (1999) found that the membrane filtration method exhibited higher recovery rates and detection limits for *Giardia* and *Cryptosporidium* compared to the cartridge filtration method. Another potential limitation of the study is the inability to identify the genotypes and species of the parasites. The rare parasitic genus *Cryptosporidium* was detected in the inlet of Mansheyat Alqanater CU using 18S rRNA amplicon sequencing, and this finding was subsequently confirmed by qPCR. While the qPCR analysis detected *Cryptosporidium* in the outlet sample, it was not detected through 18S rRNA amplicon sequencing (Table S3). This discrepancy might be attributed to the higher sensitivity of qPCR in detecting low-concentration levels.

## Conclusion

The conventional treatment processes, including coagulation, sedimentation, and filtration, have demonstrated greater effectiveness in removing *Cryptosporidium* and *Giardia* from drinking water. Specifically, neither *Giardia* nor *Cryptosporidium* was detected in conventional DWTPs, indicating the efficiency of these processes in eliminating these parasites from the source water and ensuring the safety of drinking water for consumers. Indeed, no seasonal variations for both parasites were observed. The prevalence of *Cryptosporidium* oocysts was observed to be higher than *Giardia* cysts in Nile water, suggesting a potentially higher risk of infection associated with *Cryptosporidium*, as this parasite can cause gastrointestinal illness even at low doses. The selection of technology for removing *Cryptosporidium* and *Giardia* from drinking water should be based on a comprehensive evaluation of the specific context and conditions. The presence of *Cryptosporidium* and *Giardia* cysts in CUs outlets does not automatically make this technology less effective, but rather highlights the importance of regular monitoring and maintenance practices. Ultimately, the goal should be to provide safe and reliable drinking water to consumers.

### Supplementary Information

ESM 1 (DOCX 69.3 KB)

## Data Availability

The manuscript does not contain any material from third parties, and all the material is owned by the authors, and no permission is required for publication.
