# Supplementary material for: Fate of Cryptosporidium and Giardia through conventional and compact drinking water treatment plants
Source: Parasitol Res. 2023 Aug 26;122(11):2491–501. doi: 10.1007/s00436-023-07947-8 (PMC10567834; doi:10.1007/s00436-023-07947-8)
Supplement: Supplementary file 1 — ESM 1 (DOCX 69.3 KB) [file 436_2023_7947_MOESM1_ESM.docx]

**Fate of *Cryptosporidium* and *Giardia* through conventional and compact drinking water treatment plants**

Ahmed S. Moussa^1^, Ameen A. Ashour^2^, Mohammad I. Soliman^2^, Hoda A. Taha^2^, Ahmad Z. Al-Herrawy^3^, Mahmoud Gad*^3^

^1^Reference laboratory, Drinking Water and Wastewater Holding Company, Egypt.

^2^Zoology Department, Faculty of Science, Ain Shams University, Cairo, Egypt.

^3^Environmental Parasitology Laboratory, Water Pollution Research Department, National Research Centre, 12622 Dokki, Giza, Egypt.

*Corresponding author: mi.saleh@nrc.sci.eg or mahmoudafw@gmail.com; Tel.: +20-1061132911

Figure S1. Diagrammatic view of operational steps of a conventional drinking water treatment plant.

**Clarifiers basin**

**Filters basin**

Product tank

**Pre -chlorination**

**Post chlorination**


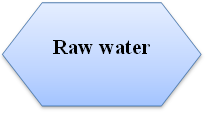


**ALUM**

**Clarification tank**

**Raw water**

**Chlorine**

**Alum**

**Filtration tank**

**Post chlorination**

**Product tank**

Figure S2. Diagrammatic view of operational steps of a compact unit.

Table S1. Total occurrence of *Giardia* cysts and *Cryptosporidium* oocysts by IFA and qPCR techniques

| **Parasite** | **IFA** | | | **qPCR** | | |
| --- | --- | --- | --- | --- | --- | --- |
|  | **Raw water** | **Treated water** | **Total** | **Raw water** | **Treated water** | **Total** |
| ***Giardia*** | 9/48  18.75% | 2/48  4.17% | 11/96  11.46% | 9/48  18.75% | 3/48  6.25% | 12/96  12.5% |
| ***Cryptosporidium*** | 14/48  29.17% | 4/48  8.33% | 18/96  18.75% | 16/48  33.33% | 4/48  8.33% | 20/96  20.83% |

Table S2. The qPCR Log_10_ values in inlets and outlets of different DWTPs and CUs, in different seasons.

| Inlet samples | | Outlet samples | | season | Station | |
| --- | --- | --- | --- | --- | --- | --- |
| qPCR_Giard | qPCR_Crypt | qPCR_Giard | qPCR_Crypt |  |  |  |
| 0 | 0 | 0 | 0 | winter | Imbaba |  |
| 2.6 | 2.4 | 0 | 0 | winter | Imbaba |  |
| 0 | 0 | 0 | 0 | winter | Imbaba |  |
| 0 | 0 | 0 | 0 | spring | Imbaba |  |
| 0 | 0 | 0 | 0 | spring | Imbaba |  |
| 2.0 | 2.5 | 0 | 0 | spring | Imbaba |  |
| 2.2 | 2.5 | 0 | 0 | summer | Imbaba |  |
| 0 | 0 | 0 | 0 | summer | Imbaba |  |
| 0 | 0 | 0 | 0 | summer | Imbaba |  |
| 0 | 0 | 0 | 0 | autumn | Imbaba |  |
| 0 | 0 | 0 | 0 | autumn | Imbaba |  |
| 2.1 | 0 | 0 | 0 | autumn | Imbaba |  |
| 0 | 2.4 | 0 | 0 | winter | Shubra Alkheyma | |
| 0 | 0 | 0 | 0 | winter | Shubra Alkheyma | |
| 0 | 0 | 0 | 0 | winter | Shubra Alkheyma | |
| 0 | 0 | 0 | 0 | spring | Shubra Alkheyma | |
| 0 | 0 | 0 | 0 | spring | Shubra Alkheyma | |
| 0 | 2.2 | 0 | 0 | spring | Shubra Alkheyma | |
| 0 | 0 | 0 | 0 | summer | *Shubra Alkheyma* | |
| 2.1 | 2.3 | 0 | 0 | summer | Shubra Alkheyma | |
| 0 | 0 | 0 | 0 | summer | Shubra Alkheyma | |
| 0 | 0 | 0 | 0 | autumn | Shubra Alkheyma | |
| 0 | 2.4 | 0 | 0 | autumn | Shubra Alkheyma | |
| 0 | 2.3 | 0 | 0 | autumn | Shubra Alkheyma | |
| 0 | 1.3 | 0 | 0 | winter | Mansheyat Alqanater | |
| 2.1 | 0 | 1.2 | 0 | winter | Mansheyat Alqanater | |
| 0 | 0 | 1.1 | 0 | winter | Mansheyat Alqanater | |
| 0 | 0 | 0 | 0 | spring | Mansheyat Alqanater | |
| 0 | 0 | 0 | 0 | spring | Mansheyat Alqanater | |
| 0 | 0 | 0 | 0 | spring | Mansheyat Alqanater | |
| 0 | 0 | 0 | 0 | summer | Mansheyat Alqanater | |
| 0 | 2.2 | 0 | 2.0 | summer | Mansheyat Alqanater | |
| 0 | 0 | 0 | 0 | summer | Mansheyat Alqanater | |
| 0 | 0 | 0 | 0 | autumn | Mansheyat Alqanater | |
| 0 | 2.7 | 0 | 1.3 | autumn | Mansheyat Alqanater | |
| 1.2 | 0 | 0 | 0 | autumn | Mansheyat Alqanater | |
| 0 | 2.2 | 0 | 0 | winter | Niklah |  |
| 0 | 0 | 0 | 0 | winter | Niklah |  |
| 2.2 | 2.5 | 1.1 | 0 | winter | Niklah |  |
| 0 | 0 | 0 | 0 | spring | Niklah |  |
| 0 | 0 | 0 | 0 | spring | Niklah |  |
| 0 | 0 | 0 | 0 | spring | Niklah |  |
| 0 | 0 | 0 | 0 | summer | Niklah |  |
| 1.4 | 1.7 | 0 | 0 | summer | Niklah |  |
| 0 | 2.2 | 0 | 1.2 | summer | Niklah |  |
| 0 | 2.4 | 0 | 1.3 | autumn | Niklah |  |
| 0 | 0 | 0 | 0 | autumn | Niklah |  |
| 0 | 0 | 0 | 0 | autumn | Niklah |  |

Table S3. 18S rRNA amplicon sequencing and qPCR for the randomly selected 8 samples of DWTPs and CUs (inlets and outlets)

| Station | Stage | 18S amplicon sequencing (no. of reads for *Cryptosporidium*) | qPCR for *Cryptosporidium* (log_10_) | 18S amplicon sequencing (no. of reads for *Giardia*) | qPCR for *Giardia*) (log_10_) |
| --- | --- | --- | --- | --- | --- |
| Mansheyat Alqanater | Inlet | 43 | 2.7 | -ve | -ve |
|  | Outlet | -ve | 1.3 | -ve | -ve |
| Niklah | Inlet | -ve | -ve | -ve | -ve |
|  | Outlet | -ve | -ve | -ve | -ve |
| Imbaba | Inlet | -ve | -ve | -ve | -ve |
|  | Outlet | -ve | -ve | -ve | -ve |
| Shubra Alkheyma | Inlet | -ve | -ve | -ve | -ve |
|  | Outlet | -ve | -ve | -ve | -ve |
